# Local Renin-Angiotensin System Activation and Myofibroblast Formation in Graft Versus Host Disease–Associated Conjunctival Fibrosis

**DOI:** 10.1167/iovs.62.13.10

**Published:** 2021-10-13

**Authors:** Kiumars Shamloo, Judy Weng, Christopher Ross, Jenni Lee, Saleh Alfuraih, Jennifer Totonchy, Ajay Sharma

**Affiliations:** 1 Chapman University School of Pharmacy, Chapman University, Irvine, California, United States; 2 Department of Pharmacology and Toxicology, Faculty of Pharmacy, Northern Border University, Rafha, Saudi Arabia

**Keywords:** GVHD, conjunctival fibrosis, conjunctival RAS, myofibroblasts

## Abstract

**Purpose:**

The present study was designed to investigate the role of myofibroblast transdifferentiation and the conjunctival renin–angiotensin system (RAS) in the pathogenesis of graft-versus-host disease (GVHD)–associated conjunctival fibrosis.

**Methods:**

A mouse model of major histocompatibility-matched allogeneic transplantation was used to induce GVHD, with male B10.D2 mice as donors and female BALB/c mice as recipients. Male BALB/c to female BALB/c syngeneic transplantation was used as control. Y chromosome staining in the spleen cells obtained from female recipient mice was used to confirm engraftment. The phenol red thread test and fluorescein staining were used to quantify tears and corneal keratopathy. Eyes were harvested at 4 and 8 weeks after the transplant for alpha-smooth muscle actin (α-SMA), angiotensinogen, and angiotensin-converting enzyme (ACE) immunostaining. Conjunctiva was harvested for gene expression quantification of α-SMA, angiotensinogen, and ACE.

**Results:**

More than 80% of the spleen cells in the recipient mice were chromosome Y positive, thus conforming successful engraftment. A significant decrease in tear secretion and a marked increase in corneal keratopathy score after allogeneic transplantation indicated the onset of ocular GVHD in these mice. A significant increase in α-SMA gene expression and the presence of a large number of α-SMA–positive cells was noted in the bulbar orbital conjunctiva of mice after allogeneic transplantation. Allogenic transplantation also caused a significant increase in the gene expression and protein expression of angiotensinogen and ACE in the subconjunctival eyelid area.

**Conclusions:**

Results of the present study demonstrate that GVHD-associated conjunctival fibrosis is accompanied by myofibroblast formation and activation of the local conjunctival RAS.

Allogeneic bone marrow transplantation is a standard treatment for many hematologic malignancies, hemoglobinopathies, and immunodeficiency diseases. However, graft-versus-host disease (GVHD) remains a challenging complication of allogeneic bone marrow transplantation. It is an immune-mediated condition that causes damage to many organs, including eyes, with 60% to 90% of patients with GVHD showing ocular complications.^1^^,^^2^ Eyes are affected in both acute and chronic GVHD, although ocular involvement is more common in patients with chronic GVHD. Acute GVHD can cause photophobia, hyperemia, conjunctivitis, lagophthalmos, and corneal ulceration,^2^^–^^5^ whereas chronic GVHD is characterized by severe ocular surface damage, resulting in dry eye, keratinization, epithelial thinning, squamous metaplasia, corneal ulceration, and meibomian gland atrophy.^2^^,^^6^

Conjunctiva is also significantly affected in patients with chronic GVHD, with such manifestations as pseudomembranous conjunctivitis, symblepharon, and fornix shortening.^2^^,^^4^ Additionally, patients with GVHD show a white scar under their bulbar conjunctiva suggestive of subepithelial conjunctival fibrosis.^7^^,^^8^ Injury to conjunctival tissue caused by a chronic inflammatory response due to GVHD can initiate a wound-healing response. An excessive wound-healing response may lead to aberrant extracellular matrix synthesis, thus resulting in conjunctival fibrosis. Myofibroblasts are contractile, metabolically active cells that synthesize large quantities of extracellular matrix proteins and play a key role in fibrosis.^9^^–^^11^ Myofibroblasts can form by transdifferentiation of a variety of cells such as resident fibroblasts, mesenchymal cells, and circulating fibrocytes.^9^^–^^11^ Conjunctiva is a connective tissue containing a significant number of fibroblasts, and the presence of donor-origin fibrocytes has also been demonstrated in the eyes of patients with GVHD.^12^

Conjunctival tissue has been shown to express components of the local renin–angiotensin system (RAS), including renin, angiotensinogen, angiotensin-converting enzyme (ACE), and angiotensin II type 1 (AT1) receptor.^13^^–^^17^ As for many other organs, angiotensin II, the end effector of the RAS pathway, has been implicated as playing a role in conjunctival fibrosis.^18^^,^^19^ Components of the RAS are upregulated after conjunctival injury.^18^ Angiotensin II has been shown to cause transdifferentiation of Tenon's fibroblasts to myofibroblasts.^19^ Profibrotic cytokines such as transforming growth factor-beta (TGF-β), platelet-derived growth factor (PDGF), and connective tissue growth factor (CTGF) that are released by immune cells in GVHD can serve as the initial trigger for fibroblast transdifferentiation to myofibroblasts.^20^^,^^21^ However, upregulation of the RAS and cross-talk between profibrotic cytokines, especially TGF-β and the RAS, has been shown to amplify and perpetuate fibrosis in non-ocular organs and could be a potential mechanism that may contribute to conjunctival fibrosis.^22^^,^^23^ The present study was designed to investigate the role of myofibroblast transdifferentiation and the conjunctival RAS in the pathogenesis of GVHD-associated conjunctival fibrosis.

## Methods

### Bone Marrow Transplantation

The animal protocol was approved by Institutional Animal Care and Use Committee of Chapman University (approval #2019-06). All animal experiments were conducted in accordance with the ARVO Statement for the Use of Animals in Ophthalmic and Vision Research. A mouse model of major major histocompatibility complex (MHC)-matched and minor MHC-mismatched mice was used to induce GVHD.^24^^–^^26^ Eight-week-old male B10.D2 mice (The Jackson Laboratory, Bar Harbor, ME, USA) having homozygous MHC haplotype d/d were used as donors, and 10-week-old female BALB/c mice (Charles River Laboratories, Wilmington, MA, USA) also having homozygous haplotype d/d were used as recipients. The control group underwent syngeneic transplantation, where donors were 8-week-old male BALB/c mice and recipients were 10-week-old female BALB/c mice. The femurs and spleen cells were harvested from the donor mice, as described in our earlier publication.^27^ Recipient female BALB/c mice were exposed to 700-cGy irradiation using an x-ray irradiator (RS2000 Series Biological Irradiator; Rad Source Technologies, Suwanee, GA, USA). After 6 hours, the irradiated recipient female BALB/c mice were injected with 2 × 10^6^ spleen cells and 1 × 10^6^ bone marrow cells. The mice were fed with a diet gel (ClearH_2_O, Portland, ME, USA), were housed in sterile cages, and received sulfatrim (0.672 mg/mL) in their drinking water for 2 weeks after the transplant. At 4 and 8 weeks after transplantation, the animals were euthanized by CO_2_ administration for the collection of ocular tissue. The study design included two different groups of mice: (1) the control group, which included BALB/c female mice (*n* = 9) that received syngeneic bone marrow and spleen cell transplantation from male BALB/c mice; and (2) the ocular GVHD group, which included female BALB/c mice (*n* =12) that received allogeneic bone marrow and spleen cell transplantation from male B10.D2 mice.

### Monitoring of Engraftment

The presence of Y chromosome in the spleen cells of female recipient mice was used to track engraftment by male donor marrow. A fluorescent paint probe was used to stain chromosome Y by fluorescent in situ hybridization (FISH) using a commercially available kit (Empire Genomics, Buffalo, NY, USA). Mice were also monitored for systemic signs of chronic GVHD, such as loss of body weight and body hair.

### Tear Quantification

Tear secretion was quantified by phenol red thread (FCI Ophthalmics, Pembroke, MA, USA) which is pH sensitive and changes from yellow to red upon wetting by tears. The thread was placed in the temporal side of the lower eyelid for 1 minute. The length of color change was measured in millimeters and converted to volume using a standard plot. A standard curve was plotted by wetting the thread with known volume of artificial tears and measuring the length of the wetted phenol red thread. Due to the small amount of tear film volume in the mouse eyes, the phenol red thread test typically requires longer time to obtain consistent values, accordingly a 1-minute duration for the phenol red thread test was used. Tear volume was quantified before the allogeneic and syngeneic transplantation (baseline) and at weeks 2, 4, 6, and 8 after the transplantation.^27^

### Fluorescein Staining

Mice were anesthetized by intraperitoneal injection of ketamine (80 mg/kg) and xylazine (5 mg/kg). A 2-µL sterile solution of 0.5% fluorescein was applied to the mouse eye for 60 seconds. The eyes were washed with 200 µL phosphate-buffered saline (PBS), and imaging was performed under a cobalt filter with a slit lamp (SL-17; Kowa USA, Torrance, CA, USA) using a digital camera. The captured corneal images were divided into four hypothetical quadrants for scoring the keratopathy using a previously published method.^27^^,^^28^ Each quadrant was scored as follows: no staining = 0; slightly punctate staining, less than 30 spots = 1; punctate staining, more than 30 spots but not diffuse = 2; diffuse staining but no positive plaque = 3; and positive fluorescein plaque = 4. The scores of each quadrant were added to arrive at a final grade (total maximum possible score = 16).

### Tissue Harvesting and Cryosectioning

Eyes along with the eyelids were harvested from euthanized animals. The tissue was fixed in 4% paraformaldehyde overnight 15% and then 30% or 15% followed by 30% sucrose. The tissue was embedded in optimal cutting temperature compound using a 2-methylbutane bath cooled over liquid nitrogen. To perform immunofluorescent staining, a cryostat (CM 1860; Leica Microsystems, Wetzlar, Germany) was used to cut sagittal sections from the frozen tissue at a thickness of 8 µm.

### Immunofluorescence Staining for Alpha-Smooth Muscle Actin, Angiotensinogen, ACE, AT1 Receptor, and Tomato Lectin Staining

Slides containing 8-µm-thick sagittal sections of the eye and eyelid were rinsed in PBS and blocked with PBS solution containing 2% bovine serum albumin for 30 minutes. The tissue sections were then incubated with primary antibodies for alpha-smooth muscle actin (α-SMA) (1:100 dilution; Invitrogen, Carlsbad, CA, USA), angiotensinogen (1:50 dilution; R&D Systems, Minneapolis, MN, USA), ACE (1:25 dilution; R&D Systems), AT1 receptors (1:50 dilution; Novus Biologicals, Littleton, CO, USA), and DyLight 594 conjugated tomato lectin (1:200 dilution; Vector Laboratories, Burlingame, CA, USA) for 90 minutes. The slides were washed with PBS three times and incubated with Alexa Fluor 488 or Alexa Fluor 647 conjugated secondary antibody (1:500 dilution; Abcam, Cambridge, UK) for 60 minutes. The nuclei were stained with 4′,6-diamidino-2-phenylindole (DAPI). The slides were imaged using a confocal microscope (Nikon, Melville, NY, USA). The number of nuclei showing α-SMA staining and the percent fraction of area showing angiotensinogen and ACE staining were quantified using Image J (National Institutes of Health, Bethesda, MD, USA).

### RNA Isolation, cDNA Preparation, and Gene Expression Quantification

Iris scissors and fine pointed forceps were used to remove the entire sheet of bulbar conjunctival tissue from the euthanized animals’ eyes at the end of 4 and 8 weeks after transplantation. The mRNA was extracted using the RNeasy Mini Kit (QIAGEN, Valencia, CA, USA). The RNA concentration was quantified using a NanoDrop spectrophotometer (Thermo Fisher Scientific, Waltham, MA, USA). The yield was between 37 and 53 ng/µL. The mRNA was immediately reverse transcribed to cDNA using a commercially available kit (SuperScript III First-Strand Synthesis System; Thermo Fisher Scientific). The cDNA was used to quantify α-SMA, angiotensinogen, and ACE gene expressions by real-time PCR. A 20-µL reaction mixture containing 2 µL of cDNA, 2 µL each of 200-nM forward primer and reverse primer (Table), and 10 µL of 2× SYBR Green Supermix (Bio-Rad Laboratories, Hercules, CA, USA) was run at a universal cycle (95°C for 10 minutes, 40 cycles at 95°C for 15 seconds, and 55°C for 60 seconds) in a thermocycler (CFX96 Touch thermocycler; Bio-Rad Laboratories). β-Actin was used as the housekeeping gene. The relative change in gene expression was calculated using the ΔΔCt method.

**Table.** Sequence of Forward and Reverse Primers Used for Real-Time PCR

Gene	Reverse Primer	Forward Primer
Angiotensinogen	5′-CAA GTT GAT CTT CCA CCC TGT C-3′	5′-TCC CAC GCT CTC TGG ATT TA-3′
ACE	5′-TTG CTG CCC TCT ATG GTA ATG-3′	5′-GAC AGG TTC GTG GAA GAG TAT G-3′
α-SMA	5′-GGC AGT AGT CAC GAA GGA ATA G-3′	5′-CCA TCA TGC GTC TGG ACT T-3′
β-Actin	5′-CCA AGA AGG AAG GCT GGA AA-3′	5′-CTC CCT GGA GAA GAG CTA TGA-3′

### Statistics

The data are presented as mean ± standard error of the mean. Statistical analysis was performed using Prism 8 (GraphPad Software, San Diego, CA, USA). The data were analyzed using two-way analysis of variance (ANOVA) for body weight, tear film volume, and corneal keratopathy and one-way ANOVA for α-SMA, angiotensinogen, and ACE gene and protein expression, followed by Dunnett's post hoc test. *P* < 0.05 was considered statistically significant.

## Results

### Characterization of GVHD and Allogenic Transplantation

**Figure 1.**
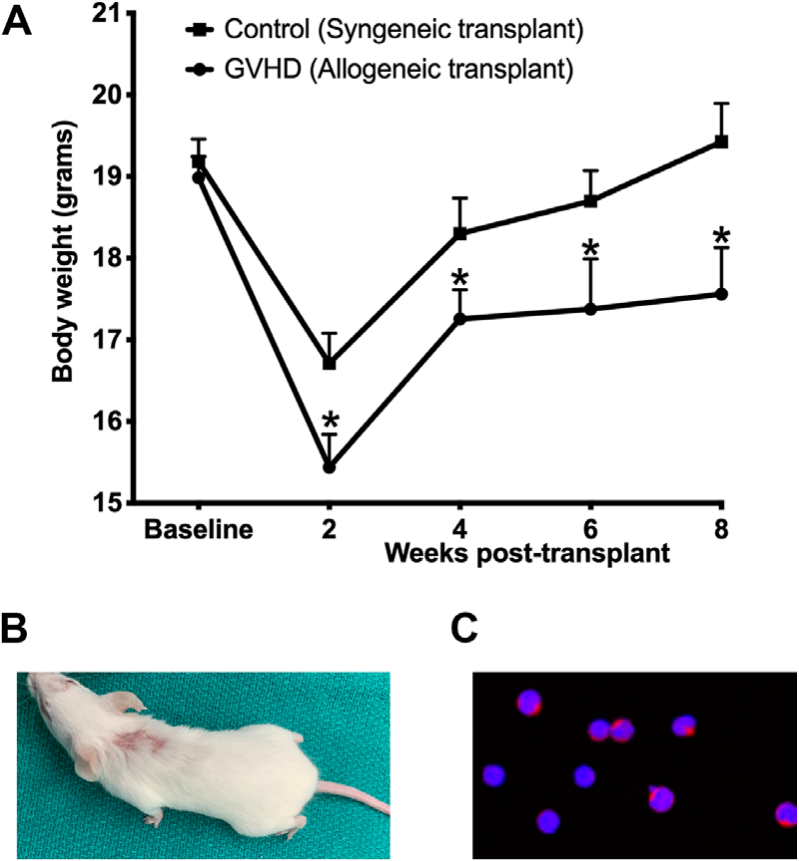
(**A**) Body weight of mice before (baseline) and at various time points after syngeneic and allogeneic transplantation. A significant decrease (*P < 0.05) in body weight was observed in mice who received allogeneic transplantation compared with mice receiving syngeneic transplantation. (**B**) Representative photograph showing signs of sclerodermatous skin lesions and hair loss in mice who received allogeneic transplantation. Spleen cells of recipient (female) mice showing Y chromosome staining confirm successful engraftment of the donor (male) graft.

Mice who received allogeneic transplantation began to exhibit signs of GVHD at around 4 weeks, including a significantly lower body weight (Fig. 1A), signs of skin scleroderma, and loss of body hair (Fig. 1B). Interestingly, a sharp decrease in body weight was observed up to the initial 2 weeks after transplantation in both groups that received either allogeneic or syngeneic transplantation. This early decrease in body weight noted in both the groups can be likely attributed to the irradiation stress (Fig. 1A). Both groups regained body weight by week 4; however, the regain of body weight was significantly lower in mice with the allogeneic transplant compared with mice that received the syngeneic transplant. Furthermore, after this initial recovery, mice with the allogeneic transplant did not show any further gain, and their body weight plateaued, suggesting the onset of systemic GVHD. In contrast, mice that received the syngeneic transplant continued to show an upward trend with a notable increase in body weight (Fig. 1A).

Because the donor mice in our model were male and the recipient mice were female, we used Y chromosome staining to confirm the successful engraftment of donor-origin marrow cells. Figure 1C shows FISH staining for Y chromosome in the spleen cells obtained from recipient female mice at 4 weeks after allogeneic transplantation. Quantification of spleen cells from five different animals indicated that an average of 80% of the spleen cells showed Y chromosome staining, thus confirming the successful engraftment of recipient mice by cells originating from male donor marrow.

### Clinical Signs of Ocular GVHD

**Figure 2.**
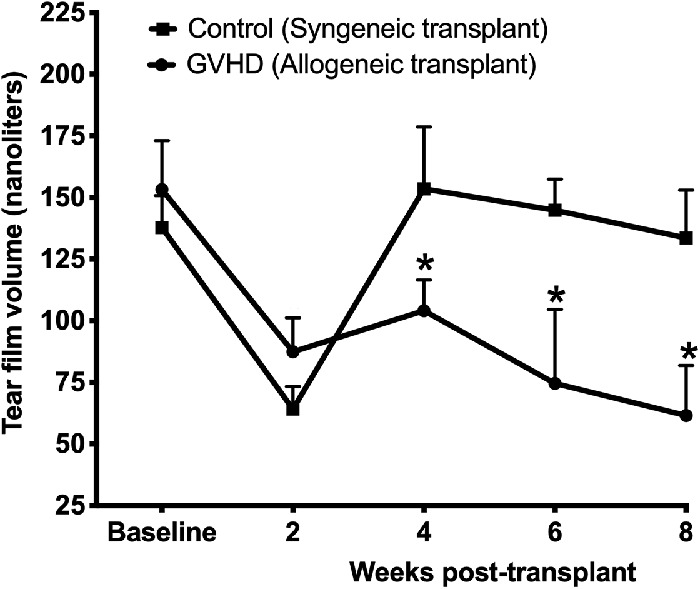
Tear film volume in mice before (baseline) and at various time points after syngeneic and allogeneic transplantation. A significant decrease (*P < 0.05) in tear film volume was observed in mice who received allogeneic transplantation compared with mice receiving syngeneic transplantation.

The mice who received allogeneic transplantation showed signs of ocular GVHD that included a decrease in tear film and onset of corneal keratopathy. Figure 2 shows the time-dependent changes in tear film volume in the mice that received either syngeneic or allogeneic transplantation. The two groups had average tear volumes of 153 nL and 137nL at baseline prior to syngeneic or allogeneic transplantation, respectively. As for body weight, both groups showed a decrease in tear film volume at 2 weeks after the transplant which could be attributed to irradiation stress-related physiological changes. However, mice that received the allogeneic transplant continued to show a further decline in tear film volume at weeks 6 and 8 after the initial slight regain at week 4. The tear volume in the mice with allogenic transplantation was significantly less compared with mice that received the syngeneic transplant.

**Figure 3.**
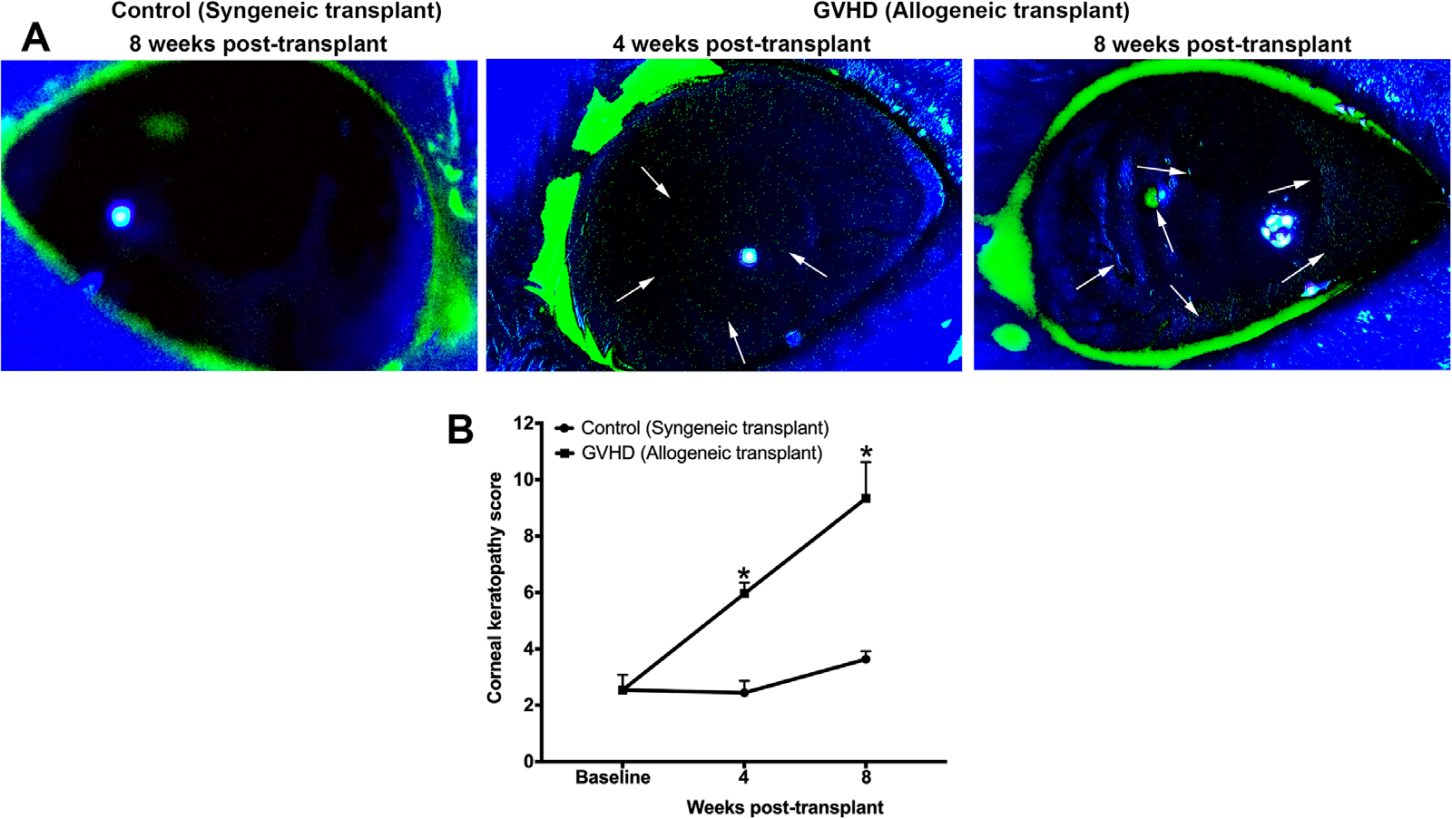
(**A**) Representative fluorescein-stained images of corneas at 8 weeks after syngeneic transplantation (control) and at 4 weeks and 8 weeks after allogeneic transplantation (GVHD). (**B**) Quantification of fluorescein staining showed significant corneal keratopathy (*P < 0.05) compared with syngeneic transplantation and before transplantation at 4 and 8 weeks after allogeneic transplantation.

Figure 3A shows representative fluorescein-stained image of mice eyes that received syngeneic or allogeneic transplantation. The corneas of mice that received allogenic transplantation showed punctate staining at week 4 and even plaques after week 8. Quantification of corneal keratopathy showed that both groups had a mean score of 2 at the baseline (Fig. 3B). Mice that received allogenic transplantation showed a significant increase in mean corneal keratopathic score to 6 and 9.3 at 4 weeks and 8 weeks, respectively. The syngeneic mice also showed a slight increase in keratopathy score to 3.6 at week 8 (Fig. 3B), possibly due to aging or irradiation, as radiation stress has been previously shown to cause corneal keratopathy.^29^^,^^30^

### Presence of Conjunctival Myofibroblasts in Ocular GVHD

**Figure 4.**
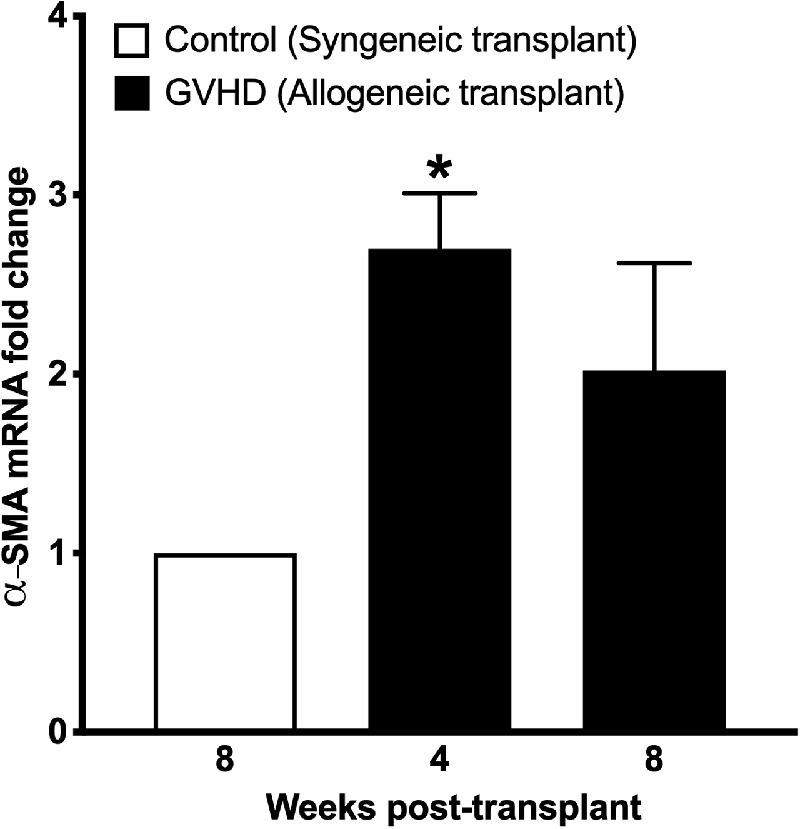
Gene expression quantification of α-SMA in conjunctival homogenates obtained from mice at 8 weeks after syngeneic transplantation (control) and at 4 weeks and 8 weeks after allogeneic transplantation (GVHD). A significant increase (*P < 0.05) in α-SMA gene expression was noted in conjunctival homogenates obtained from mice after allogeneic transplantation compared with tissues obtained from the mice that received syngeneic transplantation.

**Figure 5.**
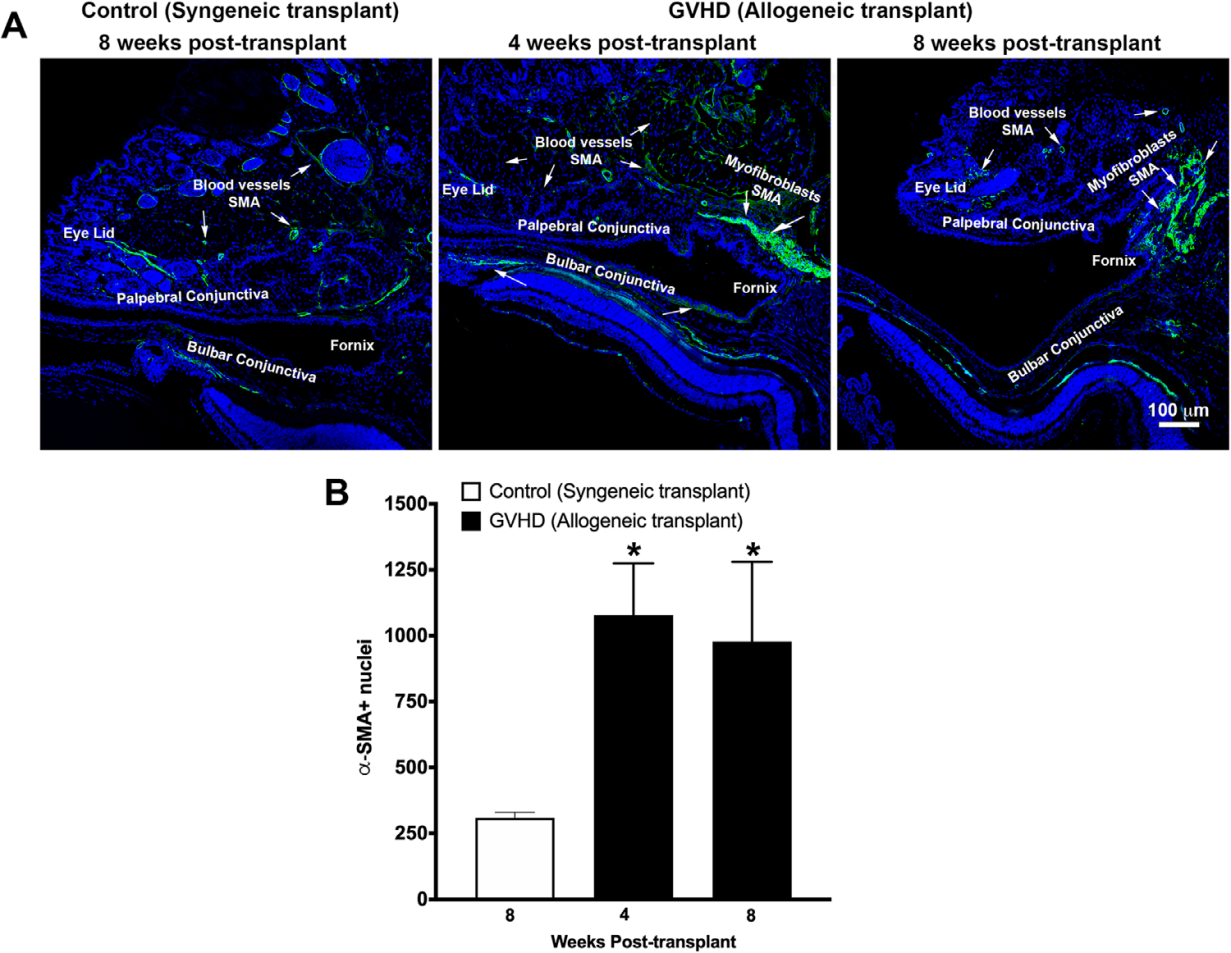
(**A**) Representative confocal images showing immunofluorescent staining (*green*) for α-SMA in eyelid tissue sections obtained from mice at 8 weeks after syngeneic transplantation (control) and at 4 weeks and 8 weeks after allogeneic transplantation (GVHD). Nuclei are stained *blue* with DAPI. (**B**) Graph shows quantification of α-SMA–stained nuclei in images obtained from five different slides each from five mice. A significant increase (*P < 0.05) in α-SMA–stained nuclei was observed in tissues obtained from mice at 4 weeks and 8 weeks after allogeneic transplantation compared with tissues obtained from mice that received syngeneic transplantation.

The presence of myofibroblasts is a hallmark feature of fibrosis. Myofibroblasts express high levels of intracellular bundles of α-SMA. Therefore, to test whether ocular GVHD causes conjunctival fibrosis due to myofibroblast formation, we used gene expression to quantify α-SMA and performed α-SMA immunostaining to detect the localization of myofibroblasts. A 2.7-fold increase (*P* < 0.05) and a 2.02-fold increase in α-SMA gene expression were observed in the conjunctival tissue obtained from mice at 4 weeks and 8 weeks after allogeneic transplantation, respectively (Fig. 4). Furthermore, immunostaining detected the presence of a large number of α-SMA–positive myofibroblasts in the bulbar orbital conjunctiva of mice at 4 and 8 weeks after allogeneic transplantation (Fig. 5A). In contrast, no myofibroblasts could be detected in the tissue sections obtained from mice that received syngeneic transplantation (Fig. 5A). Quantification of the number of nuclei showed an average of 1079 α-SMA–positive nuclei at 4 weeks and 978 α-SMA–positive nuclei at 8 weeks after allogeneic transplantation compared with around 250 α-SMA–positive nuclei in mice that received the syngeneic transplant (Fig. 5B). Eyelids are vascular tissues, and α-SMA can stain the smooth muscle actin of the vasculature. To exclude the possibility that the α-SMA staining noted in the bulbar subconjunctival space was due to the presence of vascular tissue, we performed double-staining of α-SMA with lectin (Supplementary Figure). As is evident from the double staining, lectin extensively stained the vasculature in the eyelid tissue. A double staining of lectin and α-SMA showed the typical shape of blood vessel tissue; however, α-SMA staining in the bulbar subconjunctival space did not show any lectin staining, confirming that this α-SMA staining was due to the presence of myofibroblasts (Supplementary Figure). As is evident from the nuclear staining of eyelid tissue in Figure 5A, a notable shortening of the eyelid can be seen in the histology sections obtained from the GVHD mice at 8 weeks after allogeneic transplantation. Therefore, we decided to quantify the eyelid length in the tissue sections obtained from the control and GVHD mice. Our data indicate that the length of the upper eyelid tissue sections of the control mice that received the syngeneic transplant was 1261.5 ± 26.4 µm compared with 791.5 ± 64.7 µm in the GVHD mice at 8 weeks after allogeneic transplantation. Thus, the data demonstrate that GVHD caused upper eyelid shortening in this study.

### Ocular GVHD and Conjunctival RAS Activation

**Figure 6.**
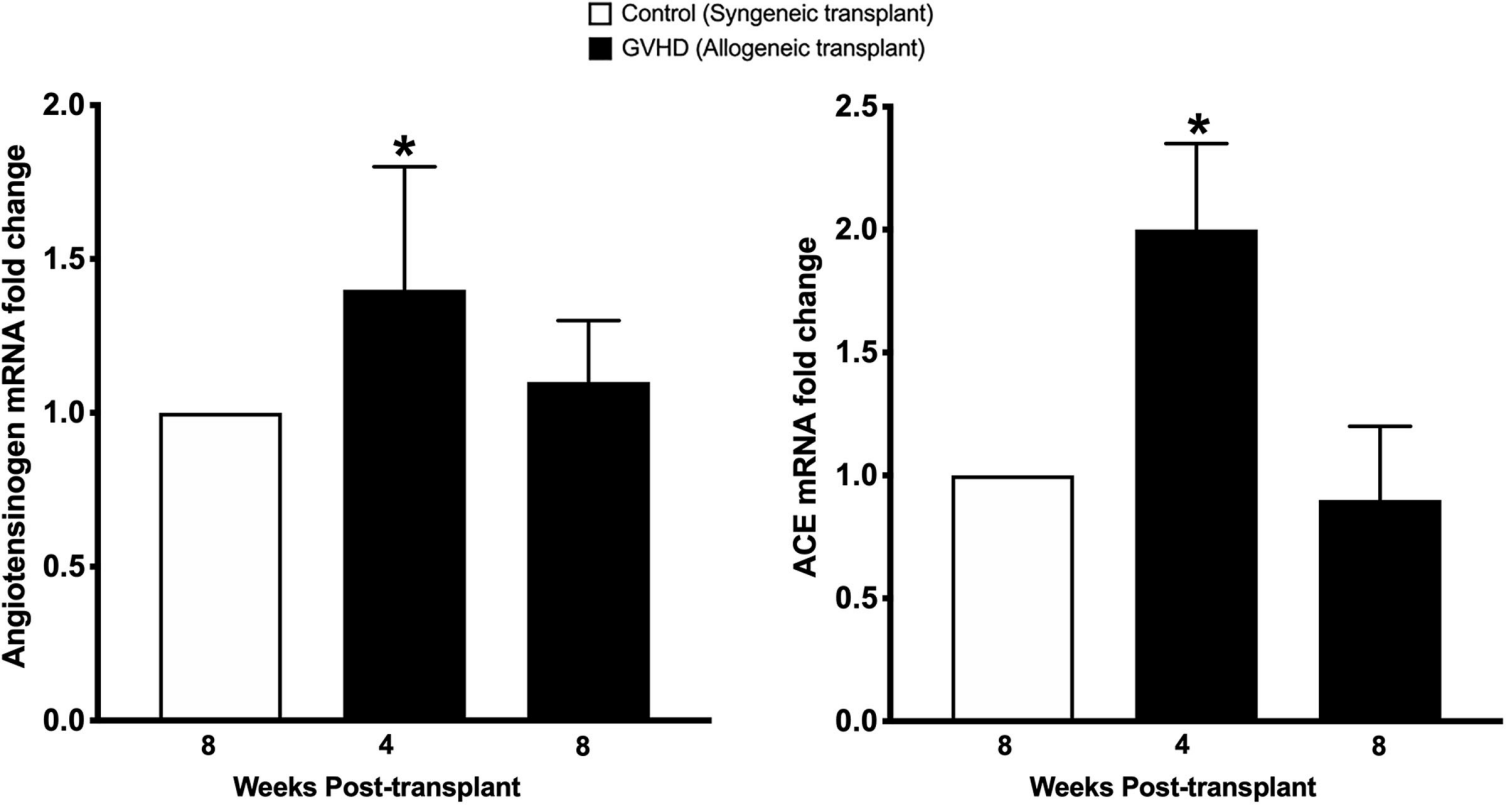
Gene expression quantification of angiotensinogen and ACE in conjunctival homogenates obtained from mice at 8 weeks after syngeneic transplantation (control) and at 4 weeks and 8 weeks after allogeneic transplantation (GVHD). A significant increase (*P < 0.05) in angiotensinogen and ACE gene expression was noted in conjunctival homogenates obtained from mice after allogeneic transplantation compared with tissues obtained from mice that received syngeneic transplantation.

To test whether conjunctival fibrosis was accompanied by activation of the local RAS, we quantified the gene expression of angiotensinogen and ACE. We also detected angiotensinogen and ACE localization by performing immunostaining. A statistically significant 1.4-fold increase (*P* < 0.05) in the gene expression of angiotensinogen and a 2-fold increase (*P* < 0.05) in the gene expression of ACE was observed in the conjunctival tissue obtained from mice at 4 weeks after allogeneic transplantation. By the 8-week time point, this increase in angiotensinogen and ACE returned to baseline (Fig. 6).

**Figure 7.**
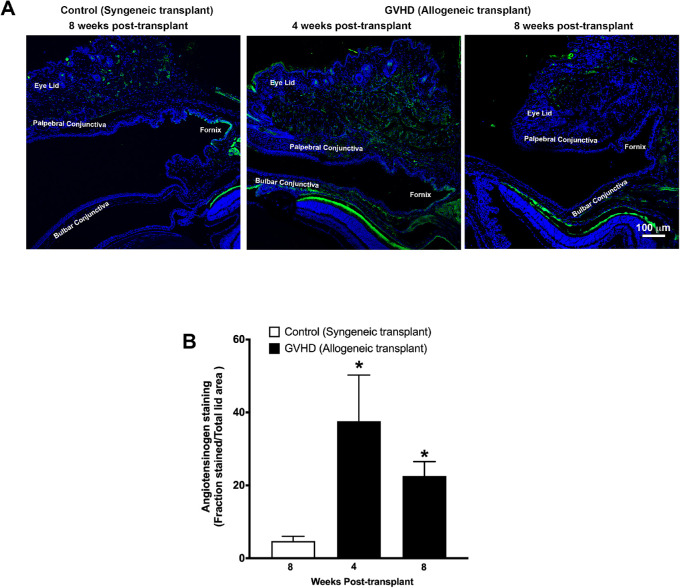
(**A**) Representative confocal images showing immunofluorescent staining (*green*) for angiotensinogen in the eyelid tissue sections obtained from mice at 8 weeks after syngeneic transplantation (control) and at 4 weeks and 8 weeks after allogeneic transplantation (GVHD). Nuclei are stained *blue* with DAPI. (**B**) Graph shows quantification of the angiotensinogen-stained area (calculated as a fraction of total eyelid area) using images obtained from five different slides each from five mice. A significant increase (*P < 0.05) in angiotensinogen-stained area was observed in tissues obtained from mice at 4 weeks and 8 weeks after allogeneic transplantation compared with tissues obtained from mice that received syngeneic transplantation.

**Figure 8.**
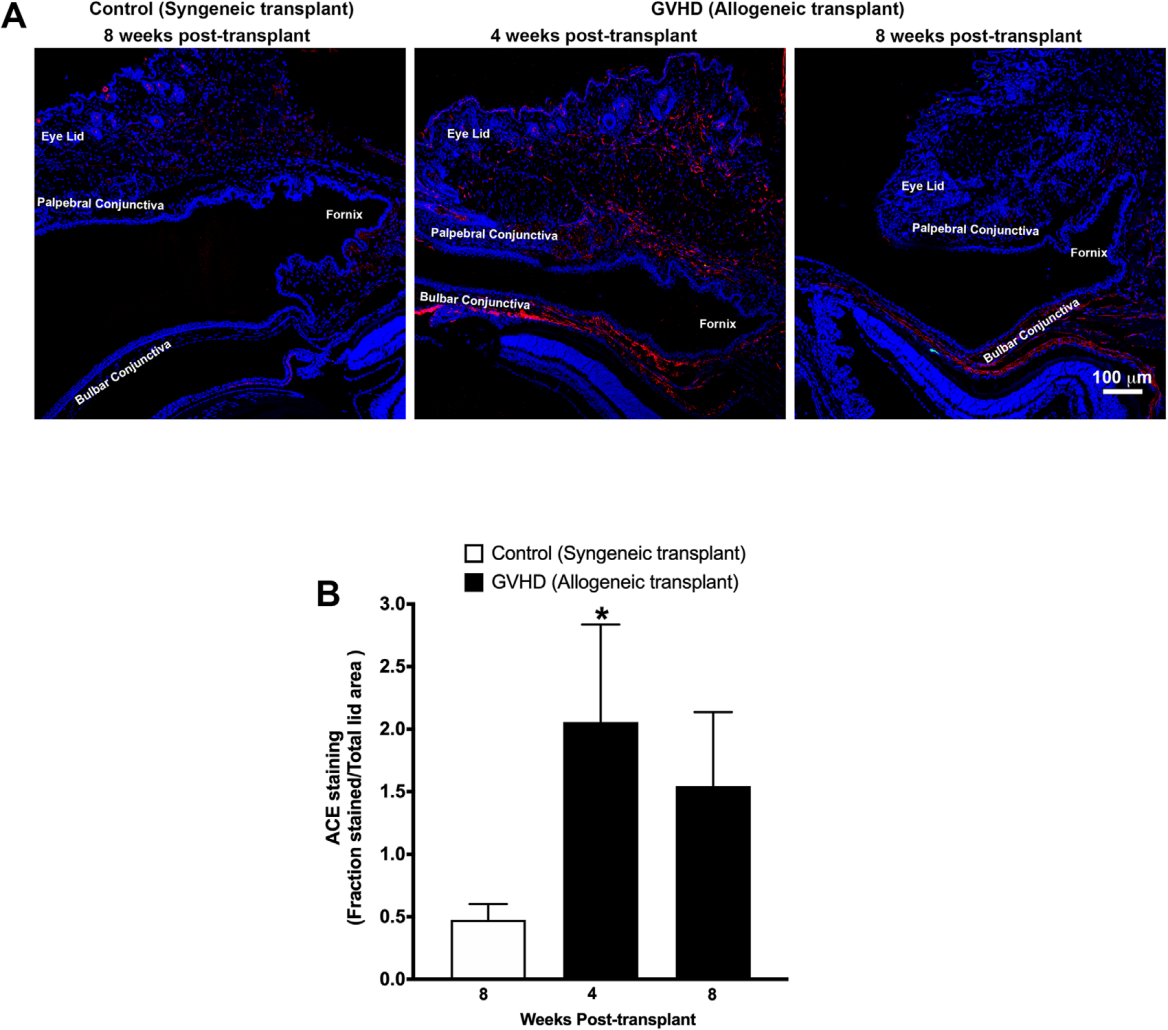
(**A**) Representative confocal images showing immunofluorescent staining (*red*) for ACE in eyelid tissue sections obtained from mice at 8 weeks after syngeneic transplantation (control) and at 4 weeks and 8 weeks after allogeneic transplantation (GVHD). Nuclei are stained *blue* with DAPI. (**B**) Graph shows quantification of the angiotensinogen-stained area (calculated as a fraction of total eyelid area) using images obtained from five different slides each from five mice. An increase in ACE-stained area was observed in tissues obtained from mice at 4 weeks (*P < 0.05) and 8 weeks after allogeneic transplantation compared with tissues obtained from mice that received syngeneic transplantation.

Immunostaining detected a large area under the bulbar conjunctiva that was intensely stained for angiotensinogen (Fig. 7A) and ACE (Fig. 8A) at 4 weeks after allogeneic transplantation. On the other hand, angiotensinogen and ACE staining was barely detectable in the eyelids of mice that received the syngeneic transplant. Quantification of percentage of stained area as a fraction of the total eyelid for angiotensinogen showed only a 4% stained area in mice that received syngeneic transplantation but 37% stained area (*P* < 0.05) and 22% stained area (*P* < 0.05) in mice at 4 weeks and 8 weeks after allogeneic transplantation, respectively (Fig. 7B). Similarly, <0.4% area showed ACE staining in mice that received syngeneic transplantation compared with 2% area (*P* < 0.05) and 1.5% area at 4 weeks and 8 weeks, respectively, in mice that received allogenic transplantation (Fig. 8B).

**Figure 9.**
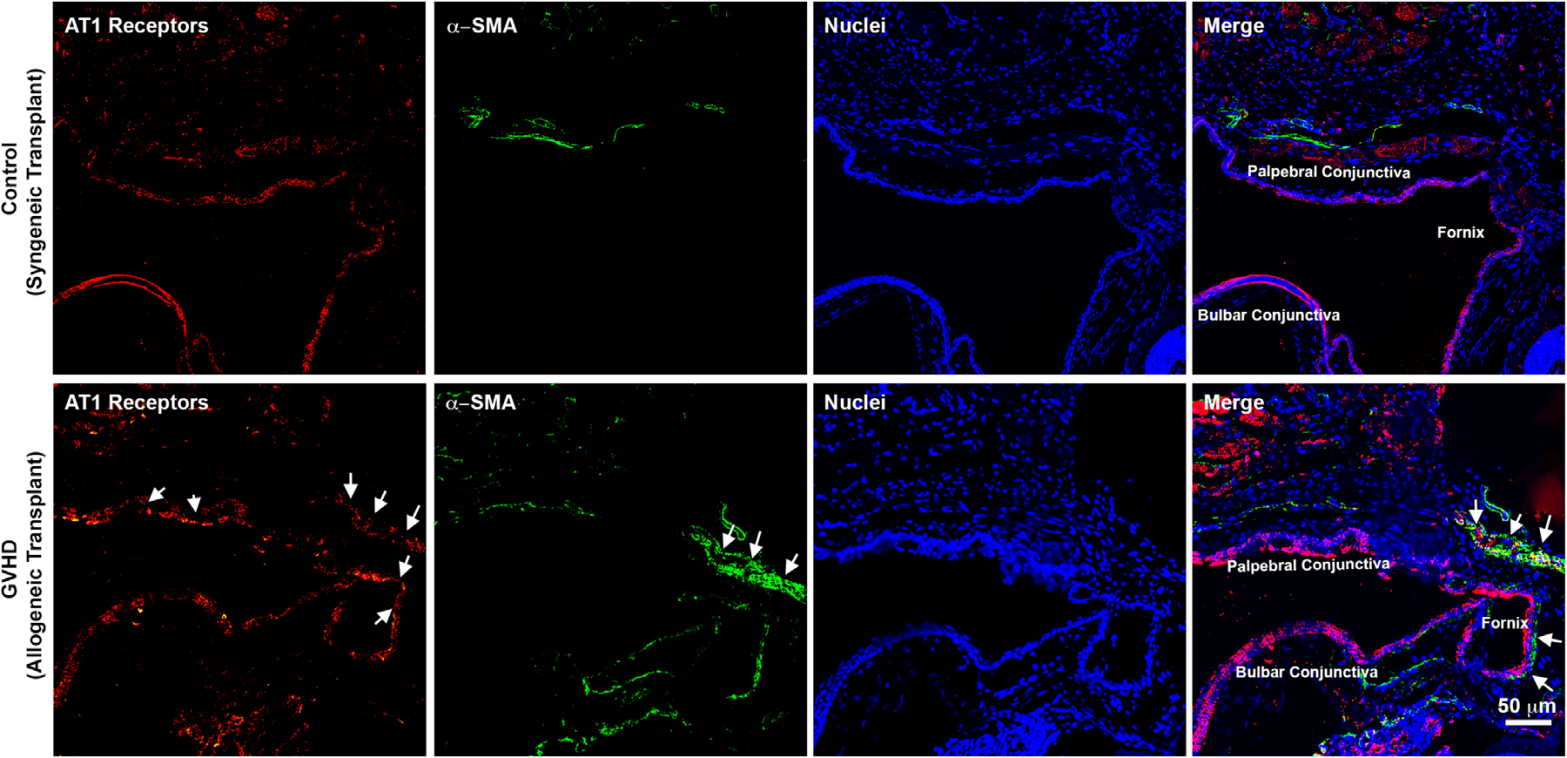
Representative confocal images showing immunofluorescent staining for AT1 receptors (*red*) and α-SMA (*green*) in the eyelid tissue sections obtained from mice after syngeneic transplantation (control) and allogeneic transplantation (GVHD). Nuclei are stained *blue* with DAPI. *Arrows* in the merge image show colocalization of AT1 receptors with α-SMA.

Finally, we examined whether AT1 receptors were expressed in the mouse conjunctiva and if there was a colocalization of AT1 receptors with the α-SMA staining. As is evident from the immunostaining images, AT1 receptor expression was detected in the conjunctival epithelium and subconjunctival space of tissue sections obtained from the control mice that received syngeneic transplantation (Fig. 9). Furthermore, a substantial increase in AT1 receptor expression was observed in the tissue sections obtained from GVHD mice that received allogeneic transplantation. It is worthwhile to note that AT1 receptor staining colocalized with the α-SMA staining in the bulbar conjunctiva around the fornix. Therefore, our data indicate that the conjunctival myofibroblasts expressed AT1 receptors (Fig. 9, merge).

## Discussion

Fibrosis is one of the key features of chronic GVHD and affects many organs, including lungs, skin, and gastrointestinal tract.^31^^–^^34^ Clinical subepithelial fibrosis appearing as a white scar has been reported in the eyelids of chronic GVHD patients.^7^^,^^8^ Studies have also shown a correlation between conjunctival fibrosis and corneal epitheliopathy, suggesting that conjunctival fibrosis likely contributes to dry eye and ocular surface damage in GVHD.^7^^,^^8^ The present study used a B10.D2 to BALB/c major MHC-matched and minor MHC-mismatched mouse model to investigate whether this mouse model shows features of conjunctival fibrosis as characterized by the presence of myofibroblasts. This mouse model exhibits scleroderma-like features, a finding confirmed in the present study, and the model has been extensively used to study GVHD-associated fibrosis in various organs.^24^^–^^26^ The results of the present study demonstrate that this model indeed shows features of conjunctival fibrosis as demonstrated by the presence of myofibroblasts. Furthermore, the model recapitulates many other features of ocular GVHD, including dry eye and corneal keratopathy. Interestingly, conjunctival fibrosis was noted at 4 weeks without any further increase in severity at 8 weeks, whereas the tear film and keratopathy score continued to decline up to the tested time point of 8 weeks. This trend tentatively suggests that the conjunctival fibrosis sets in early and may contribute to the ocular surface damage in this model that has been observed in clinical studies.

The repair process during fibrosis typically involves the deposition of excessive extracellular matrix which results in replacement of normal parenchymal tissue with the hypertrophic scar tissue. Myofibroblast formation and proliferation are critical steps in fibrosis.^9^^–^^11^ Myofibroblasts are metabolically active cells that synthesize and secrete large quantities of extracellular matrix during fibrosis.^9^^–^^11^ Myofibroblasts also contribute to the altered mechanical properties of tissues because they express high amounts of contractile bundles of the cytoskeletal protein α-SMA. The present study demonstrated a large number of α-SMA–positive myofibroblasts in the orbital bulbar conjunctiva, suggesting that GVHD-associated conjunctival fibrosis is accompanied by myofibroblast formation. The noted increase of α-SMA gene expression at week 4 after transplantation followed by a decline at week 8, but a comparable number of α-SMA-stained cells, suggests an initial proliferative phase of myofibroblast formation after allogeneic transplantation followed by a stabilization phase. The resolution phase of fibrosis is typically characterized by a decline in the myofibroblast number due to apoptosis and other mechanisms. Interestingly, results of the current study demonstrate that the resolution of GVHD-associated conjunctival fibrosis likely requires more time, as a significant number of myofibroblasts were still present in the conjunctiva of the GVHD mice up to the tested time point of 8 weeks. Furthermore, a shortening of the eyelid was also consistently observed in the eyes of the GVHD mice at 8 weeks. Given the detection of large populations of myofibroblasts and their localization in the bulbar orbital conjunctiva of the GVHD mice, it is highly likely that the tissue contractile changes caused by these myofibroblasts may be contributing to the noted alterations in the eyelid architect of these GVHD mice.

GVHD is an immune condition mediated by a complex interplay between donor immune cells and host tissue. Immune cells of donor origin have been shown to be present in the recipient conjunctival tissue.^35^ These immune cells can release a variety of profibrotic cytokines such as TGF-β, PDGF, and CTGF that can trigger fibroblast activation and their transdifferentiation to myofibroblasts.^36^^–^^40^ Further, these immune cells and activated fibroblasts also secrete profibrotic vasoactive peptides such as endothelin and angiotensin that can perpetuate a vicious cycle of excessive wound healing and fibrosis.^36^^–^^40^ Activation of the RAS and elevated levels of its effector molecule angiotensin II have been shown to play a critical role in mediating fibrosis in liver, skin, lung, and cardiac tissue.^41^^–^^45^ Ocular tissue has been shown to express all components of the RAS.^13^^–^^17^^,^^37^ Overaction of the RAS has been shown to play a critical role in conjunctival fibrosis after trabeculectomy.^18^^,^^19^ Results of the present study demonstrate that GVHD-associated myofibroblast formation was accompanied by a significant increase in the gene and protein expression of angiotensinogen and ACE in the tissue around the conjunctiva. Moreover, the presence of AT1 receptors was also detected in the eyelids of the GVHD mice, and the receptors colocalized with α-SMA staining, suggesting that myofibroblasts in conjunctival tissue express AT1 receptors. Angiotensin II directly acting on these AT1 receptors or through increased levels of TGF-β can promote fibroblast proliferation and myofibroblast differentiation. Therefore, the noted increase in local angiotensinogen and ACE activity observed in this study is likely to be an important contributor to the initiation and perpetuation of conjunctival fibrosis and myofibroblast formation. Overlapping temporal kinetics between the concomitant increase in angiotensinogen and ACE and the increase in α-SMA expression, as noted in the present study, further support this hypothesis.

In conclusion, results of the present study demonstrate that GVHD-associated conjunctival fibrosis is accompanied by myofibroblast formation and activation of the local tissue RAS.

## Supplementary Material

Supplement 1
