# Supplementary material for: Local Renin-Angiotensin System Activation and Myofibroblast Formation in Graft Versus Host Disease–Associated Conjunctival Fibrosis
Source: Invest Ophthalmol Vis Sci. 2021 Oct 13;62(13):10. doi: 10.1167/iovs.62.13.10 (PMC8525838; doi:10.1167/iovs.62.13.10)
Supplement: Supplement 1 [file iovs-62-13-10_s001.pdf]

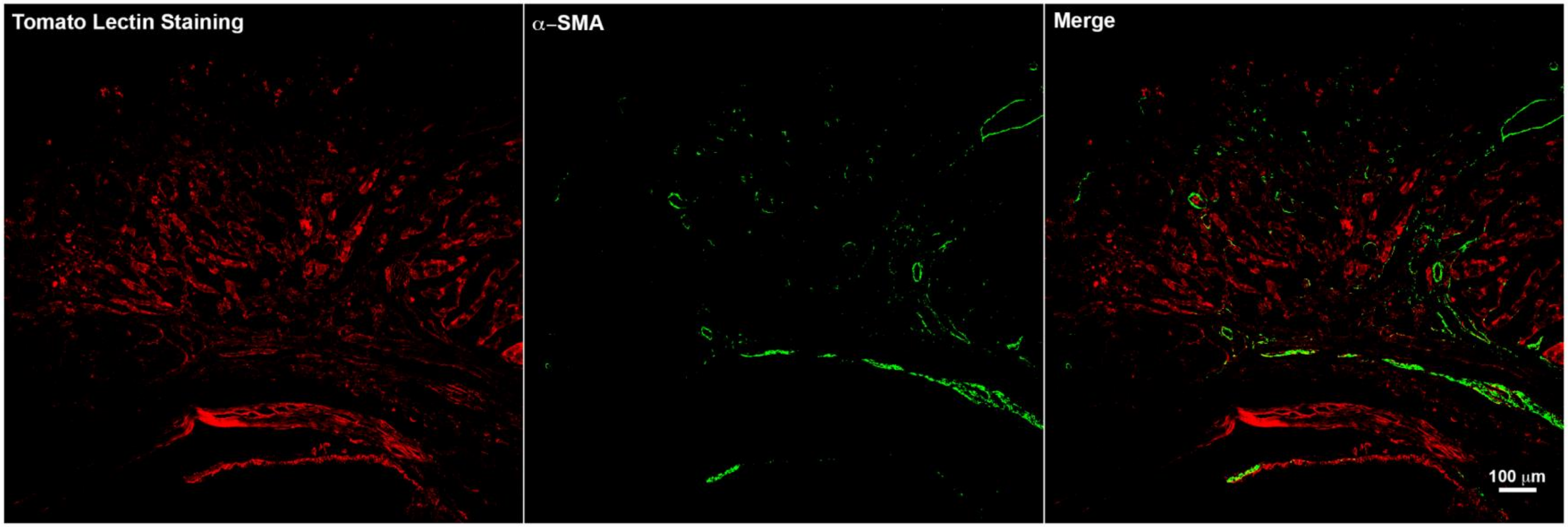

Supplement Figure: Representative confocal images showing immunofluorescent staining of vasculature using tomato lectin (red) and α-SMA (green) in the eyelid tissue sections obtained from mice after allogeneic transplant (GVHD).
